# Assessing Ecological Risks from Atmospheric Deposition of Nitrogen and Sulfur to US Forests Using Epiphytic Macrolichens

**DOI:** 10.3390/d11060087

**Published:** 2019-06-03

**Authors:** Linda H. Geiser, Peter R. Nelson, Sarah E. Jovan, Heather T. Root, Christopher M. Clark

**Affiliations:** 1 Water, Wildlife, Fish, Air & Rare Plants Directorate, Forest Service, U.S. Dept. of Agriculture, 201 14th St SW, Mailstop 1121, Washington, DC 20250, USA; 2 Penobscot Experimental Forest, Northern Research Station, Forest Service, U.S. Dept. of Agriculture, and University of Fort Kent, Maine, 54 Government Road, Bradley, ME 04411, USA; 3 Pacific Northwest Research Station, Forest Service, U.S. Dept. of Agriculture, 620 SW Main St., Suite 502, Portland, OR 97205, USA; 4 Department of Botany, Weber State University, 1415 Edvalson St., Dept. 2504, Ogden, UT 84408-2505, USA; 5 National Center for Environmental Assessment, Office of Research & Development, U.S. Environmental Protection Agency, 1200 Pennsylvania Ave. NW, Washington, DC 20460, USA

**Keywords:** critical load, lichen, air pollution, environmental assessment, quantile regression, ecological risk, climate, biodiversity, conservation, land management, CMAQ, PRISM

## Abstract

Critical loads of atmospheric deposition help decision-makers identify levels of air pollution harmful to ecosystem components. But when critical loads are exceeded, how can the accompanying ecological risk be quantified? We use a 90% quantile regression to model relationships between nitrogen and sulfur deposition and epiphytic macrolichens, focusing on responses of concern to managers of US forests: Species richness and abundance and diversity of functional groups with integral ecological roles. Analyses utilized national-scale lichen survey data, sensitivity ratings, and modeled deposition and climate data. We propose 20, 50, and 80% declines in these responses as cut-offs for low, moderate, and high ecological risk from deposition. Critical loads (low risk cut-off) for total species richness, sensitive species richness, forage lichen abundance and cyanolichen abundance, respectively, were 3.5, 3.1, 1.9, and 1.3 kg N and 6.0, 2.5, 2.6, and 2.3 kg S ha^−1^ yr^−1^. High environmental risk (80% decline), excluding total species richness, occurred at 14.8, 10.4, and 6.6 kg N and 14.1, 13, and 11 kg S ha^−1^ yr^−1^. These risks were further characterized in relation to geography, species of conservation concern, number of species affected, recovery timeframes, climate, and effects on interdependent biota, nutrient cycling, and ecosystem services.

## 1. Introduction

### 1.1. Air Pollution As A Concern of Natural Resource Managers

Sustaining the diversity, health, and productivity of natural resources now and into the future are common mission elements of regulatory, land management, and other governmental agencies. Air quality is a natural resource of particular interest as evidenced by the many laws and policies guiding its protection and assessment in the US (Clean Air Act, Wilderness Act, National Forest Management Act, National Environmental Policy Act, Federal Lands Policy and Management Act), the 56 countries of the UNECE (under the Convention on Long Range Transboundary Air Pollution [1]), and countries of the United Nations [2]. Because air pollution impacts biological diversity, ecosystem health, and associated ecosystem services, such as clean water, food, and fiber [3–6], it can impede the accomplishment of mission-related goals for managing land and protecting the environment. Therefore, tools that can help assess the risk of environmental harm from air pollution are of direct interest to managers, regulators and policy-makers. Here we focus on a subset of air pollutants of particular consequence to environmental health: Sulfur (S)- and nitrogen (N)-containing eutrophying and acidifying pollutants.

### 1.2. Air Pollution as a Human Health Concern

Air pollution, notably in the form of fine particulates (PM_2.5_), is also an important concern to human health and well-being. It is directly linked to impaired visibility and a range of acute and chronic human health effects, such as myocardial infarction, hypertension, congestive heart failure, arrhythmias, cardiovascular mortality, chronic obstructive pulmonary disease and lung cancer, diabetes, decreased cognitive function, attention-deficit or hyperactivity disorder and autism in children, and neurodegenerative disease, including dementia, in adults. Worldwide it is a leading cause of early mortality and has major socio-economic costs related to lost productivity and increased expenditures on health care [7,8]. Recent studies strongly suggest that some health effects can occur at close to background levels of fine particulate air pollution [9]. Therefore, protecting air quality benefits both human health directly and the environment and ecosystem services upon which humans depend.

### 1.3. Critical Loads: Thresholds of Harm from Atmospheric Deposition

Critical loads (CLs) of atmospheric deposition are a tool that can help assess whether a given level of pollution is likely to cause environmental harm. A CL is defined as “the quantitative estimate of an exposure to one or more pollutants below which significant harmful effects on specified sensitive elements of the environment do not occur according to present knowledge” [10]. Critical loads are calculated for specific receptors, such as forest soils, surface waters, or vegetation, often using a dose-response relationship. Critical loads are typically expressed as loading rates (as opposed to ambient concentrations of pollutants under a certain size), such as kilograms (kg) or equivalents of N and S per hectare per year [11].

### 1.4. Lichen Critical Loads Provide Broad Environmental Protection

Lichens have a long history as key biological indicators of harm from air pollution, [12,13]. As one of the most sensitive components of forested ecosystems, shifts in lichen community composition provide an overall indication of air quality for forest health and productivity [14,15]. Studies in the US and Europe have demonstrated that levels of N and S deposition tolerated by lichens are often tolerated by other biological receptors [16–18]. Thus, preventing the exceedance of lichen CLs can provide broad protection to the terrestrial ecosystem.

We recently calculated national scale lichen CLs for the atmospheric deposition of N and S in US forests [19]. These CLs (1.5 kg N ha^−1^ y^−1^ and 2.7 kg S ha^−1^ y^−1^) prevent pollution-driven shifts in community composition of epiphytic macrolichens and were applicable under all current climate regimes. Above these deposition levels, community composition increasingly favored the presence of tolerant species over sensitive ones, a response long-recognized in lichen-N studies [20–22].

While these CLs may be broadly protective of forest vegetation, how would a manager or regulator evaluate the ecological risk accompanying their exceedance within a particular site or management unit? And more directly, how would different levels of air pollution impact core mission goals? We answer these questions using lichens as a model receptor and the US EPA’s framework for ecological risk assessment [23,24], below:

### 1.5. Assessing Ecological Risks from Exceedance of Lichen Critical Loads

**Figure 1.**
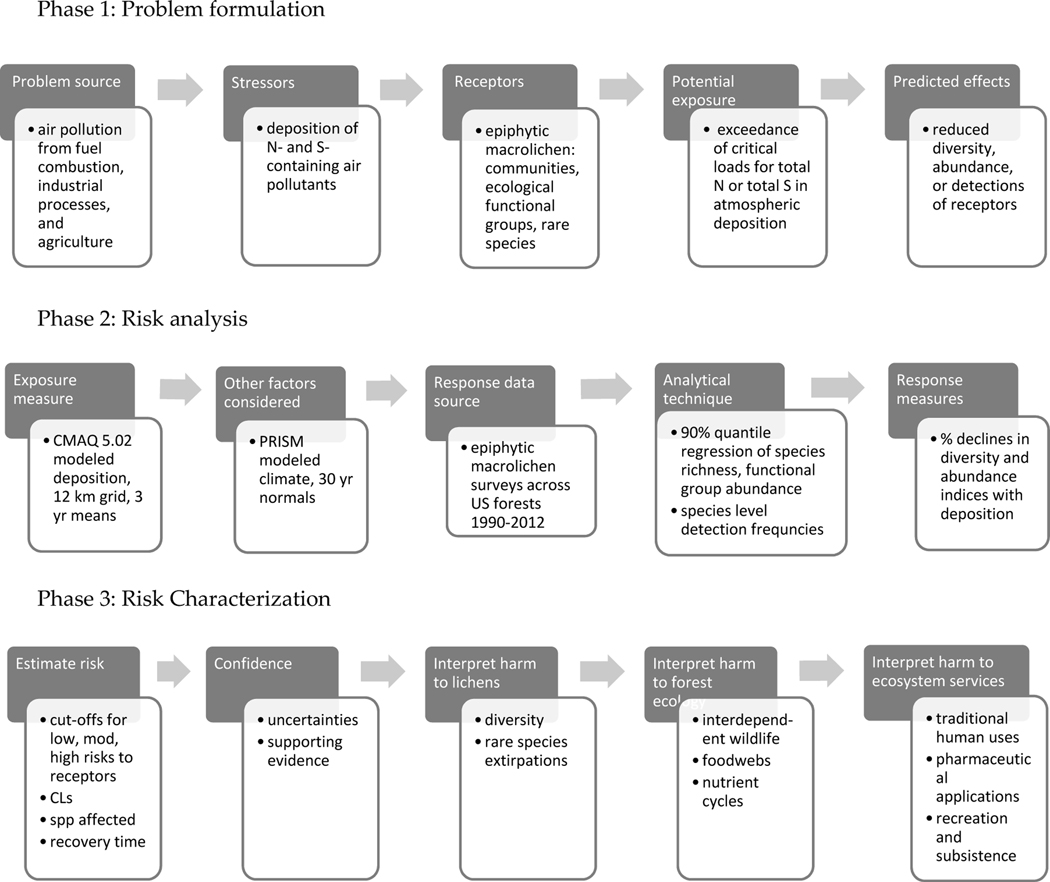
Process for assessing the ecological risk posed by the effects of air pollution on forest lichens used in this report.

Figure 1 outlines our adaptation of the EPA’s framework for this study. Phases 1, 2, and 3 are covered in the Introduction, Methods and Results, and Discussion, respectively. The evolution of critical loads science and application in the US has been an iterative process involving many discussions and workshops between researchers, managers, and policy-makers, e.g., [4,25]. This new approach has been informed by these on-going interactions.

#### 1.5.1. Phase 1: Problem Formulation

In problem formulation, we respond to the concerns of managers and policy-makers regarding situations where CLs for lichen receptors are exceeded—namely, how to predict and interpret environmental harm to forest resources and services affected by lichen decline. Specifically, we identify the stressors, receptors, and effects of pollution exposure relevant to mission imperatives and legal responsibilities of land managers (e.g., [26–29]) to:

1. Conserve and promote biodiversity.
2. Sustain or enhance ecosystem integrity, productivity, and services.
3. Prevent extirpations of rare and conservation concern species.

#### 1.5.2. Phase 2: Risk Analysis

**Figure 2.**
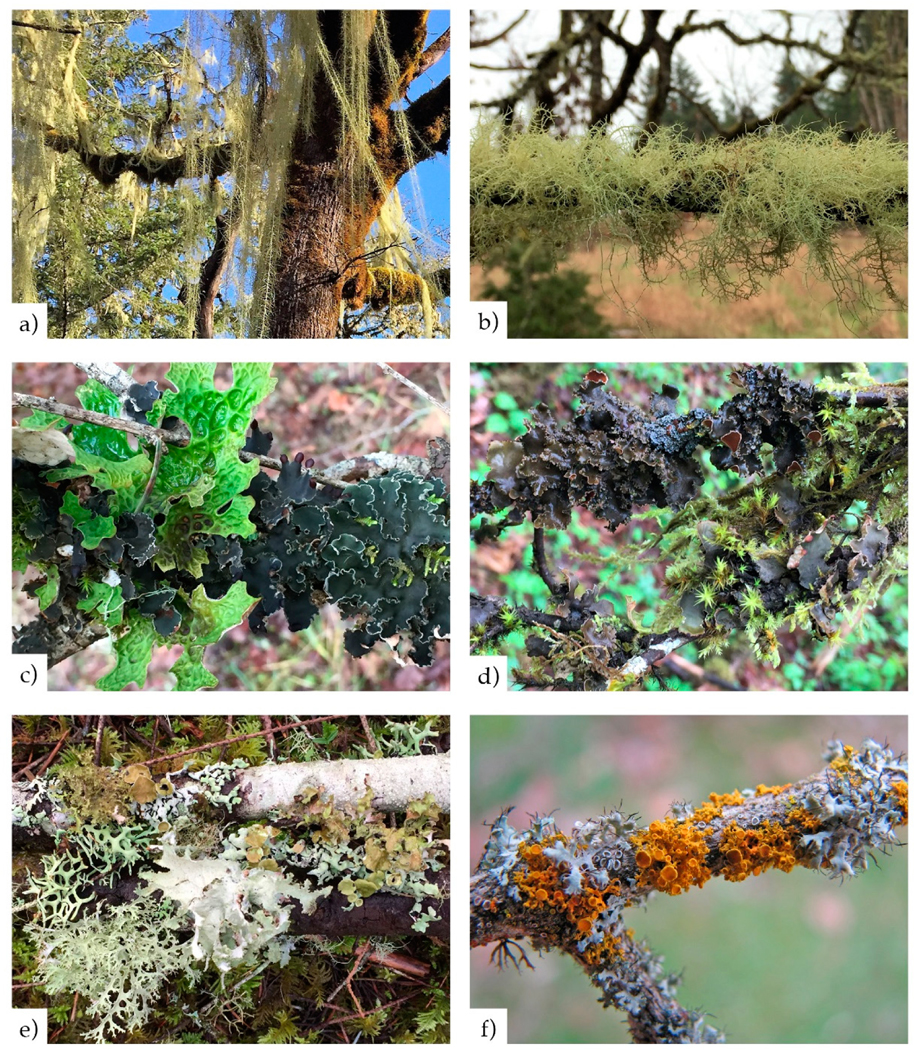
Epiphytic macrolichens exemplifying (**a**) pendant forage lichens, (**b**) shrubby forage lichens, (**c**) large cyanobacterial lichens (cyanolichens), (**d**) small to medium cyanolichens, (**e**) medium to large matrix lichens, and (**f**) small matrix lichens. Near Philomath, western Oregon. Photo credit: (**f**) Jim Riley.

Risk analysis means determining the degree to which the selected receptors are exposed to deposition and the likelihood of subsequent harmful effects. We derived 6 metrics to evaluate risk at a given site within the three focal categories above:

1. Total species richness (α-diversity) and sensitive species richness. Species counts are a direct measure of biodiversity within a site.
2. Diversity and abundance of key ecological functional groups: Forage, cyanobacterial, and matrix lichens. See Table 1 and Figure 2 for ecological roles and illustrations of genera assigned to these groups.
3. Detection frequency of individual species of conservation concern—building on the data and species level sensitivity ratings from our previous study [19].

We used 90% quantile regression to model relationships between metrics and atmospheric deposition, which provided predictive equations for determining % decline at a given deposition level. Climate is another major driver of lichen communities [31–33] and so modeling accounted for potential effects multiple climate variables on metrics. Methodological and analytical uncertainties were assessed.

#### 1.5.3. Phase 3: Risk Characterization

To evaluate and describe risks, we quantify deposition levels associated with 20, 50, and 80% declines in the lichen metrics. We follow up with a discussion of the number of species affected, time frames of effects, and risks to interdependent biota and forest nutrient cycles. We also discuss impacts on ecosystem services, such as traditional and pharmaceutical uses of lichens, the production of food and fiber, recreation, and subsistence lifestyles.

## 2. Materials and Methods

Sections 2.1 and 2.2 summarize lichen and environmental data described in detail by [19] while Sections 2.3–2.7 build upon their analyses. Their data, including taxonomic decisions (Table S1), splines of species’ detection frequencies vs. deposition, and N and S sensitivity ratings for 362 species (Table S2) are permanently archived at [30]. Data and R-code for the new analyses presented here are also archived in this location.

### 2.1. Lichen Data

#### 2.1.1. Lichen Surveys

Lichen surveys of epiphytic macrolichen communities were conducted by the US Forest Service from 1990–2012 at 8855 sites, mostly following the Forest Inventory & Analysis (FIA) Lichen Communities Indicator protocol [34]. Plots fell roughly on a 27 km^2^ grid, with intensified sampling in some locations to represent urban forests and protected areas, such as designated wilderness. Survey data consisted of a list of species detected by a trained observer on the survey site, typically a 0.38 ha circular plot, and the ocular abundance codes recorded on site for each species—1 (1 to 3 detections); 2 (3–10 detections); 3 (10 detections to occurrence on up to half of available substrates; or 4 (the species occurs on more than half of available substrates). Lichen identities were verified by lichen specialists following [35].

#### 2.1.2. Air Pollution Sensitivity Ratings

Species sensitivity ratings by [19] were quantitative and equal to the total deposition of N or S (in kg ha^−1^ y^−1^) at each species’ maximum detection frequency. Splines were used to examine species’ detection frequencies in the eastern and western US as a function of deposition estimates from the Community Multi-scale Air Quality (CMAQ v. 5.02) model (see Section 2.2.1). The Great Plains is a broad, non-forested, natural boundary separating eastern from western forests. Thus, each species could have up to 4 ratings (N and S in the East and West). To be rated, a species had to have at least 8 detections and display an interpretable response to deposition (decreasing, hump-shaped, or increasing); 362 species were rated, ~18% in both East and West.

#### 2.1.3. Assigning Species to Functional Groups

We assigned each species to one of six functional groups based on morphology, physiology, and ecology: Large (1) and small- to medium-sized (2) cyanobacterial lichens (cyanolichens); pendant (3) and shrubby (4) fruticose green algal lichens (forage lichens); and medium to large (5) and small (6) foliose green-algal lichens (matrix lichens). Table 1 summarizes lichen ecological roles by functional group; photos of example species are provided in Figure 2. Divisions into similar functional groups have been found useful for interpreting climate and pollution responses by others [36–41].

### 2.2. Environmental Data

#### 2.2.1. Deposition Data

For the continental US, total N and S deposition were extracted from 12 km Community Multiscale Air Quality modeling system (CMAQ v.5.02) gridded output [42] overlaid on-site coordinates (as 3-year means ending on the survey year.) For Alaska coastal forests, where CMAQ output was unavailable, deposition estimates relied on lichen element concentrations calibrated to through-fall deposition (for N) [43] or to CMAQ (for S) [19].

The deposition data source is fundamental to the resulting CLs and risk cut-offs. Deposition data from different models or on-site measures vary in their associated uncertainties and chemical species assessed and are not necessarily directly comparable. Uncertainties associated with the CMAQ deposition estimates [44–46] include uncertainties in emissions inventories, completeness of the chemical budgets (especially organic N), representation of chemical reactions, deposition algorithms, and any spatial or temporal resolution limitations (e.g., missing spatial hotspots of deposition from orographic effects [47]). Additional uncertainty may arise from systematic or unsystematic uncertainty in empirical measurements used to bias-adjust or train these models [42].

Recently, ref [46] published a detailed analysis of uncertainty in various forms of N and S deposition from CMAQ v.5.0.2 by comparing modeled estimates with measurements of wet deposition from NADP sites and measurements of concentration (for dry deposition) from CASTNET sites. Bias was not reported for concentrations or dry deposition, but correlations were generally high (R ranged from 0.88 to 0.95). Overall, their assessment suggested that our estimates of total N and S deposition in the eastern US may be lower than what is experienced at FIA plots. This could have resulted in an underestimate of CLs here. As models and emissions inventories improve, lichen CLs should be recalculated; however, at this time, the CLs reported here were derived from the best available information.

#### 2.2.2. Climate Data

Thirty-year climate normals for mean annual precipitation, mean maximum August temperature, and mean minimum December temperature, and continentality (the difference between the August and December values) were extracted from the Parameter-elevation Regressions on Independent Slopes Model (PRISM) [48,49] for survey site coordinates.

Cut-offs for normals were the 4th and 5th year of the decade, e.g., 1970–2000 normals were applied to 1995–2004 surveys. Climatic moisture deficit (CMD) [50] was generated from 1961–1990 climate normals using ClimateNA v5.10, http://tinyurl.com/ClimateNA. PRISM data for 1980–2018 time series studies using climatologically-aided interpolation have recently become available [51]. We recommend this dataset for future assessments of lichen community response to the changing climate in the continental US.

### 2.3. Calculating Lichen Metrics

#### 2.3.1. Biological Diversity

Species richness or α-diversity provides a metric for the biological diversity of epiphytic macrolichens at each survey site. The FIA protocol is not ideal for estimating total species richness because surveys have a 2 h time limit and are usually conducted by non-specialists [52]. However, surveyors typically have botany training and are certified by professional lichenologists before conducting the method. Furthermore, the same protocols are applied at each plot, so even though true species richness is usually somewhat higher than the estimated species richness, the two measures are correlated [53]. Surveyors collect any individuals they think might be different species, which helps boost the diversity captured. The minimum QA requirement is that surveyors detect at least 65% of the species observed by a professional lichenologist in the same time period [34]. Studies using FIA data show that estimates from different observers for the same plot typically vary by 35% even when minimum data quality standards are met [52,53] with actual performance varying by surveyor and sub-region [54]. Use of the 90% quantile should reduce variability, due to observer error by favoring the best collectors.

#### 2.3.2. Indices of Abundance for Forage, Matrix and Cyanolichen Functional Groups

Metrics were needed for the ecological contributions of the functional groups at a given site. We considered that lichens fulfill their ecological roles best (e.g., providing plentiful forage, nesting materials, or insect habitat) when they are both diverse and abundant—but mainly when they are abundant, e.g., [55–57]. Therefore, we excluded species with abundance codes of 1 or 2 (<15 detects per 0.38 ha). Given the 2-hour limit and large survey area, it is easier to detect species that are large, abundant, and showy versus small, rare, and cryptic. Therefore, we expect that focusing on species rated as 3’s and 4’s increased the repeatability of this metric by eliminating the main driver of differences in species richness estimates, the detection of rare and uncommon species [52]. Additionally, 4’s should, roughly speaking, have an order of magnitude more biomass than 3’s [36], although look-alike forage lichens can be difficult to document as 4s because of the number of close inspections required. After experimenting with several arithmetic and logarithmic indices, we selected the sum of species abundances greater than 2. Thus, the site value for three species of cyanolichens of abundance ‘3’ and two species with abundance ‘4’, would be 18 (3 + 3 + 3 + 4 + 4). The larger the metric value, the greater the presumed ecological value of the functional group at the site. Indices based on summing abundance ratings are common in other studies using FIA data, e.g., [32,33].

#### 2.3.3. Diversity of S- and N-Sensitive Lichens

The sensitive lichens metric was the richness of sensitive species detected on the site. Species were considered sensitive if the sensitivity rating (deposition at peak detection frequency in Table S2 from [19,30] was < 4.2 kg N or < 2.7 kg S ha^−1^ y^−1^. These cut-offs correspond to minimum deposition levels for the East; a practical decision that allowed eastern ‘decreasers’ to be rated sensitive, while minimizing the inclusion of intermediately tolerant species from the west with ‘hump-shaped’ responses to deposition. The higher the value for this metric, the greater the value of the site for supporting air pollution sensitive lichens. To emphasize their different species compositions, the N-sensitive species were called ‘oligotrophs’. Like the total species richness metric, this metric includes species from all the functional groups and with any abundance rating.

### 2.4. Statistical Analyses

#### 2.4.1. Comparing Air Pollution Sensitivities of Species among Functional Groups

To assess air pollution effects on the ecological roles played by lichens, an important consideration is the distribution of sensitivities within a functional group. A broad distribution would presumably assure that at least some species would be sufficiently abundant to carry out the group’s roles across a broad range of atmospheric deposition. A group that consists only of sensitive species would be less able to carry out the group’s roles as deposition increased beyond optimal ranges for those sensitive species. Another consideration was the diversity of species within functional groups. Presumably the more species in a functional group, the more robust the ecological function under stress—whether from air pollution or climate. Thus, our main questions were: (1) Are there significant differences in the distribution of sensitivity ratings among functional groups (i.e., sensitivity means and variances) and if so, which groups are different? (2) Do any groups lack tolerant species? (3) How many species are present in each functional group and does the proportion of species in each group differ between the western US and eastern US?

Because [19]’s species sensitivity ratings could not be lower than the minimum or higher than the maximum deposition in the region, the distributions of sensitivity ratings had heavy tails. We used JMP11.2.0 [58] statistical software’s non-parametric Analysis of Means test to compare means of functional groups (as transformed ranks) to the overall mean for lichen sensitivities. Analysis of Means for Variances-Levene Absolute Deviance from the Median (ADM) was used to compare variances [59]. Eastern and western sensitivity ratings were analyzed separately for N and for S and also plotted as quantile distributions. Total species diversity within each functional group, specifically γ-diversity [60] for eastern and western regions, was assessed from the respective total species counts. A t-test was used to compare relative proportions of species counts in the east vs. west.

Finally, to determine whether rare species were more likely to be pollution-sensitive than common species, we compared means and variances (as above) of rarely detected species (detected at <1% of sites in the East or West) to those of commonly detected species (detected on >10–40% of sites in Table S2 from [19]). This definition of ‘rare’ refers to regional presence/absence distribution and should not be confused with plot level ocular abundance ratings.

#### 2.4.2. Rationale for 90% Quantile Regression as a Modeling Approach

When plotting the lichen metrics for survey sites as a function of deposition, we noticed a consistent pattern. Maximal responses peaked at the lowest deposition value and decreased in a curvilinear pattern with increasing deposition. However, a wide range of less than maximal responses also occurred at every deposition level (to visualize this, see data points in Figures 5 and 6, represented by green and orange circles for western and eastern sites, respectively). We interpreted this pattern as follows: Maximal responses were limited primarily by air quality, whereas non-maximal responses could be caused by limitations in suitable climates, substrates, habitats, proximity to reproductive propagules, observer performance, etc. The 90% quantile was selected to represent the response to deposition alone under otherwise optimum environmental and survey conditions in the data set. This quantile is resistant to outliers, does not assume equal variance across the range of responses and is more stable across the wide range of conditions compared to the mean [61]. In contrast, the mean response, as is often the focus of in regression models, is more influenced by the average conditions across plots, including limitations caused by other predictors that are not of particular interest here. The 90th quantiles of the five lichen community metrics could then be modeled with increasing deposition and/or climate, as described below.

#### 2.4.3. Modeling Response of Lichen Metrics

Each lichen metric was regressed against the predictors using the package ‘quantreg’ [62] in R [63]. Hypothesizing that deposition was the single best predictor of lichen metrics, we built full models for S and N, including all climate variables and reduced models of sole deposition, individual climate variables, or all climate variables. Models for deposition-only were tested with additional polynomial terms. Since the models were nested, the Akaike information criterion (AIC) was calculated to compare the models for each metric. The largest change in AIC was used to select the best model (Δ AIC > 25).

#### 2.4.4. Assessing Model Reliability

To measure the model fit, we used a statistic called R1 [64], which ranges from 0 to 1. It is calculated as one minus the ratio of the absolute residuals between a model with predictors and the intercept (numerator) divided by a model with only the intercept (denominator). This is different than r^2^, which compares the sum of squares from the fitted line. R1 weights the sum of the point distances from the fitted function based on the quantile function (90th percentile in this case). Model fit improves (R1 increases) as the residual error for the ‘predictors + intercept’ model decreases relative to the residual error for the ‘intercept only’ model. We also calculated a bootstrapped 95th percentile confidence interval for the fitted line [65] replicating model fit 10,000 times.

### 2.5. Quantifying Critical Loads and Risk Classes

Deposition values at 0, 10, 20, 50 and 80% declines in the 90% quantile for each metric were calculated heuristically from model equations. Declines of 0–20% were considered low risk and the 20% value was selected as the critical load. Declines of 50% and 80% were the cutoffs for moderate and high risk, respectively. Declines >80% were considered to be very high risk. (See Section 4.2.1 for rationale). Model equations were used to calculate declines in indices associated with incremental increases of S and of N deposition from 1 to 20 kg ha^−1^ y^−1^. Finally, counts or abundances were calculated for 0, 10, 20, 50 and 80% declines in the 90% quantile for each lichen metric.

### 2.6. Assessing Extirpation Risk for Species of Conservation Concern

What if a manager wishes to evaluate individual species’ risks of extirpation from air pollution on a given land unit? The first step is to determine the species of concern from local lichen survey data or applicable lists of species conservation status. If the pollution sensitivities of the species are rated in Table S2 [19], the values in that table can be used to assess extirpation risk. The risk can be quantified as anticipated declines in detection frequency up to 20% (low), up to 50% (moderate), up to 90% (high), or by more than 90% (very high) compared to peak detection frequency. We provide an example in Section 3.3 using 12 randomly selected species that were rare in our national dataset (i.e., detected at >8 sites, but < 1% of sites or < 22 times in the eastern US or < 67 times in the western US; number of detections in the national dataset from Table S2 [19]).

We caution that risk estimates based on shifts in the community or functional group composition are more robust than those for individual species. If a species distribution is limited to climates where deposition is always low or always enhanced, then the rating is less certain compared to species that are distributed across broader climatic and deposition gradients. The principle strengths of individual species ratings are that they encompass the species’ national distributions, are based on systematic surveys using standardized survey protocols, and, Alaska excepted, are evaluated using a single model for deposition data. Thus, they represent the best data that has been available to date.

### 2.7. Mapping Lichen Metric Values

Values for each lichen metric were mapped across all survey sites using JMP 11.2.0 statistical software [58] to visualize national patterns of lichen diversity and abundance; and to provide perspective for regional and across-site comparisons. Color gradients were scaled across maps to match deposition and climate minima and maxima in [19].

## 3. Results

### 3.1. Variability in Air Pollution Sensitivity among Functional Groups and Rare Species

**Figure 3.**
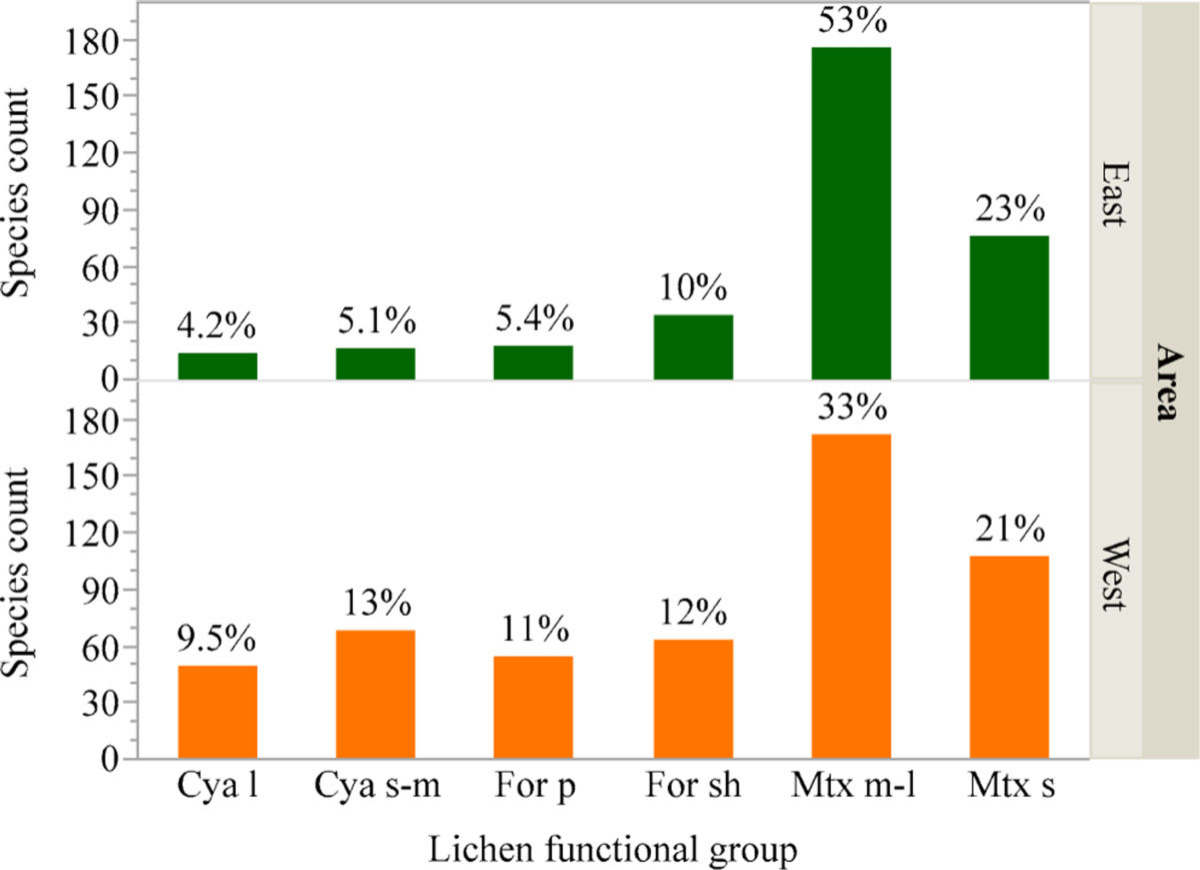
Species richness of matrix lichens was similar in the eastern and western US, but species richness of forage and cyanolichens was higher in the west. Forage and cyanolichens also comprised a higher percentage of western, compared to eastern, species. Values above bars indicate the percentage of species in that functional group for the East or West. Abbreviations: Cya = cyanolichen, For = forage lichen, Mtx = matrix lichen, l = large, s-m = small to medium, sh = shrubby, s = small, m-l = medium to large.

**Figure 4.**
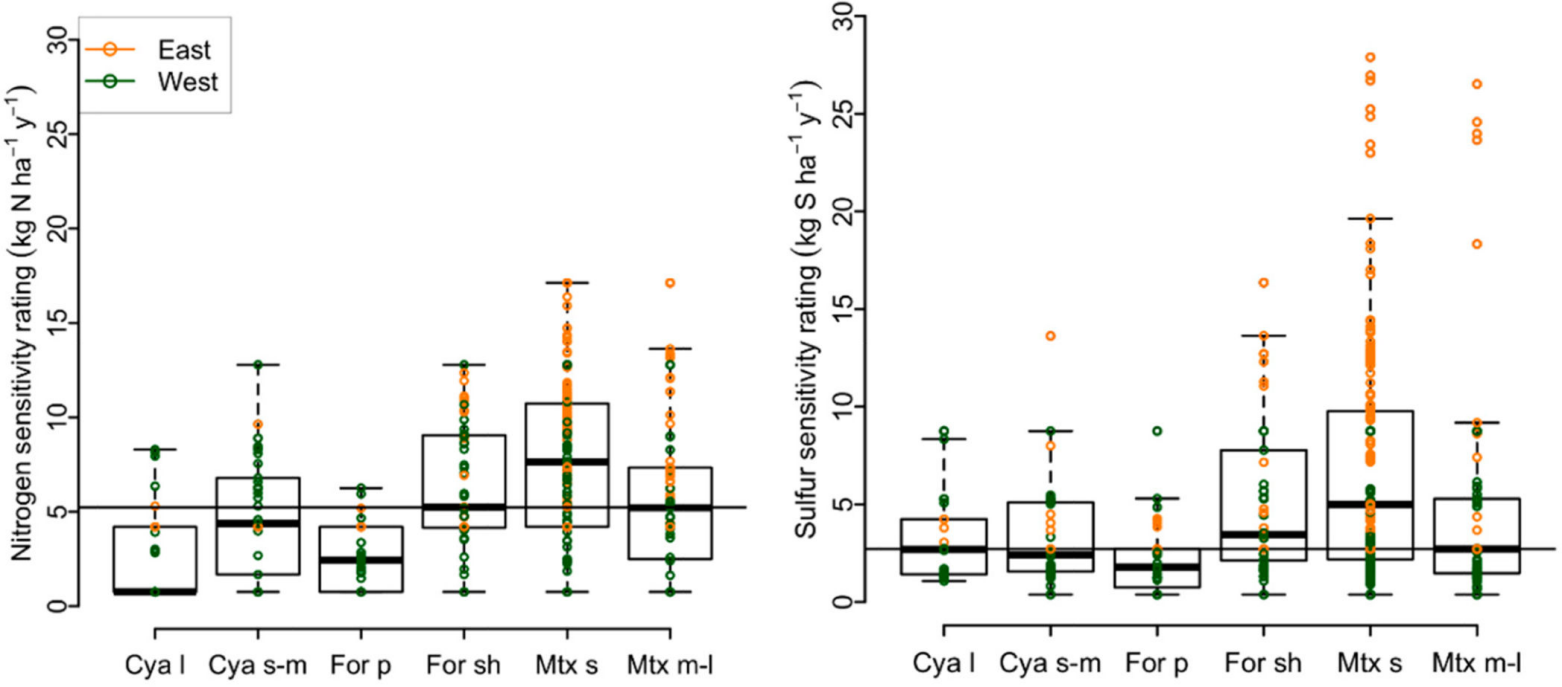
Box and whisker plots of lichen sensitivity ratings by functional group. Boxes enclose 25–75% quantiles, whiskers mark upper and lower outliers (1.5 times the interquartile range). The horizontal line indicates the overall median for the pollutant. Very few cyanolichens or pendant forage lichens were tolerant to S or N (sensitivity rating > 8 kg ha^−1^ y^−1^). Medium to large matrix lichens exhibited the broadest range of sensitivities. Abbreviations as in Figure 3.

**Figure 5.**
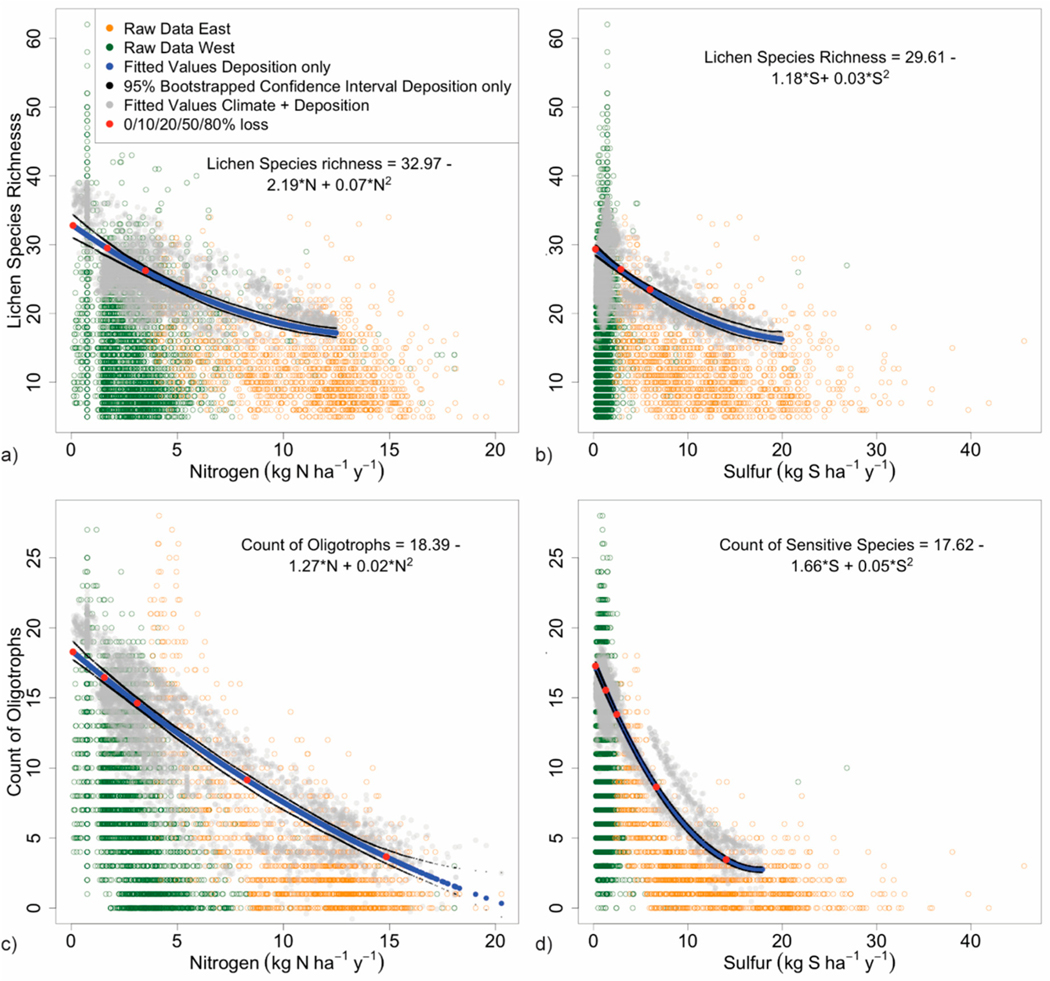
90% quantile regression of deposition vs. (**a**) total species richness for N, (**b**) total species richness for S; and (**c**) oligotroph species richness for N, and (**d**) S-sensitive species richness for S. Western and eastern sites are denoted by green and orange circles, respectively. Blue lines represent the fitted line for the 90% quantile predicted by the deposition only model; bootstrapped 95% confidence intervals are in black. Red dots indicate declines in the metric of 0, 10, 20, 50, and 80%. Fuzzed gray dots indicate 90% quantiles for the deposition + climate model.

**Figure 6.**
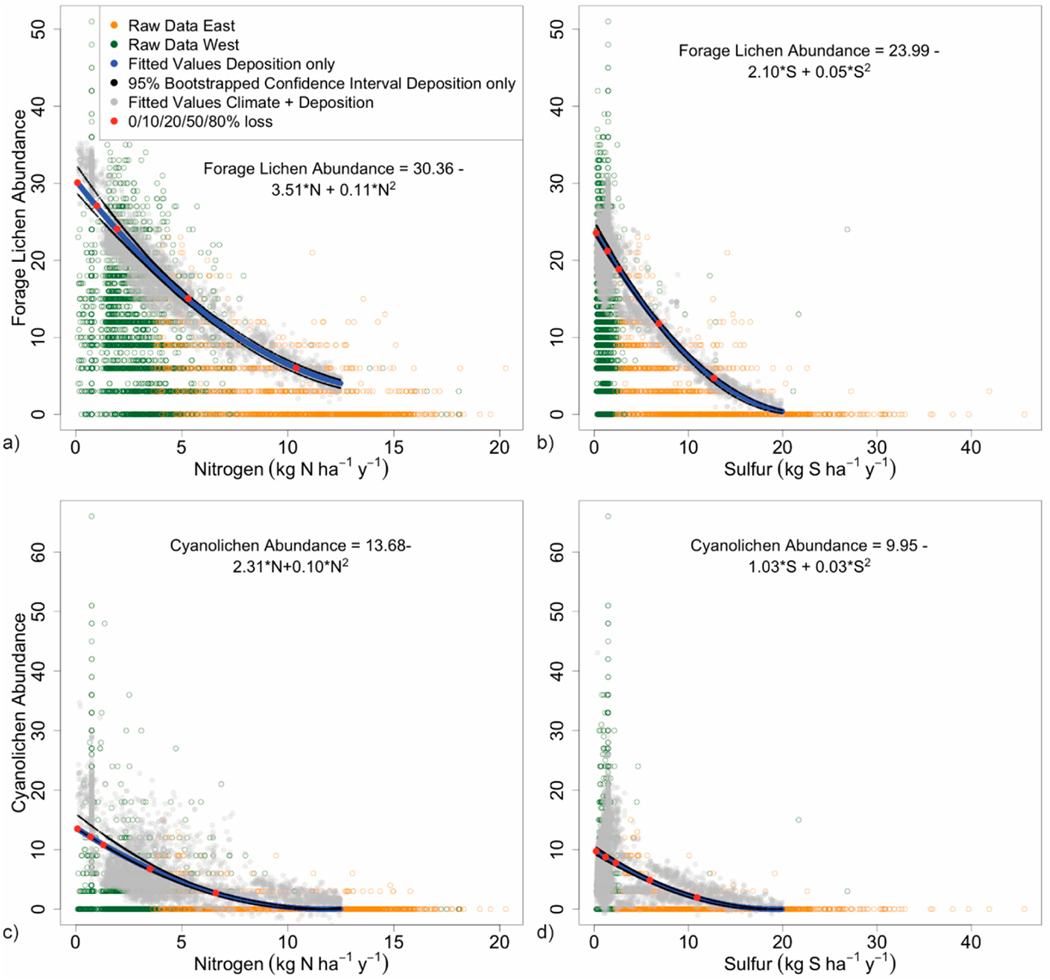
90% quantile regression of deposition vs. forage lichen diversity and abundance for (**a**) N and (**b**) S, and of deposition vs. cyanolichen diversity and abundance for (**c**) N, and (**d**) S. Western and eastern sites are denoted by green and orange circles, respectively. Blue lines represent the fitted line for the 90% quantile predicted by the deposition only model; bootstrapped 95% confidence intervals are in black. Red dots indicate declines in the metric of 0, 10, 20, 50, and 80%. Fuzzed gray dots indicate 90% quantiles for the deposition + climate model.

Large cyanolichens and pendant forage lichens had lower species richness and comprised a smaller proportion of the total flora compared to other groups (Figure 3). These functional groups encompassed few tolerant species, were on average more sensitive to deposition, and exhibited a narrower range of species sensitivities, particularly for N (Figure 4; i.e., lower limits exceeded group means in analyses of means and variances tests for N, *p* < 0.05). By contrast, medium to large matrix lichens had highest species counts, comprised more than half the flora, were on average more tolerant of air pollution with many tolerant species, and exhibited a wider range of sensitivity ratings compared to other groups (Figure 4; upper limit exceeded group means in analyses of means and variances tests for N in the east, *p* < 0.05). Shrubby forage lichens, small to medium cyanolichens, and small matrix lichens were generally intermediate to the other functional groups. Forests of the East and West supported similar matrix lichen species counts (252 and 279), but four-fold more cyanolichens (117 vs. 31) and twice as many forage lichens (118 vs. 52) were encountered in the West (Figure 3). For the latter groups in the East, air pollution is implicated as a major limiting factor, consistent with higher deposition there (Figures 5 and 6). Mean sensitivities and variances of rare species to N and S deposition did not differ from those of common species, except in the West where the mean for S-sensitive rare species was lower than that of common species (Tables S1,S2).

### 3.2. Response of Lichen Metrics to Deposition

Optimized responses of each lichen metric, represented by the 90% quantile regression of deposition alone, exhibited a negative curvilinear relationship to increasing deposition (Figures 5 and 6). Predicted values for lichen metrics from ‘deposition + climate’ models overlaid on the fitted line and bootstrapped confidence intervals for ‘deposition only’ models illustrate variability contributed by climate to the 90% quantile. We modeled most responses only up to 12 kg N and 20 kg S ha^−1^ y^−1^ because there were insufficient data from higher deposition locations.

The large Δ AIC values in Table 2 demonstrate that adding deposition to climate only N or S models (Dep + Clim) greatly improve the model over the combined climate variables (Clim) alone, indicating a strong role of deposition in the response across nested models. Absolute model fit (R1) of ‘deposition only’ models ranged from 0.07 to 0.26 and, except for S cyanolichens, were always higher than ‘climate only’ models. Bootstrapped 95% confidence intervals were tight for all models, indicating very little variability in model fit along the deposition gradient. Matrix lichen R1 values were 0 for both S and N models had R1’s of 0 and were not further analyzed.

The equations for the ‘deposition only’ models (Figures 5 and 6) were used to calculate declines in the 90% quantile associated with deposition (Tables 3 and 4). We selected ‘deposition only’ models over the ‘deposition + climate’ models for several reasons. The deposition was nearly always a better predictor by itself than the combined climate variables and we were most interested in how deposition alone limited maximum potential response given otherwise suitable environmental conditions, including suitable climates. Calculating separate risk factors for all possible combinations of climate and deposition at the scale of the entire country was beyond the scope of this paper. We could have held climate constant in the models, but that would also not have represented the wide variability in climate. For the ‘deposition only’ models, the cyanolichen metric appeared the most pollution sensitive, followed by forage lichens, oligotrophs, S-sensitive lichens, and total species richness. For additional measures of model fit and multicollinearity of predictors, see Tables S3 and S4. These tables show Pearson’s correlations among the individual deposition and climate predictors and provide variance inflation factors: calculated as the ratio of variance in the ‘climate + deposition’ model, divided by the variance of a model with each of the climate predictors alone. Mean maximum August temperature and mean annual precipitation were the most influential of the climate predictors.

#### 3.2.1. Species Richness

Across the national N and S deposition gradients, 90% quantiles for species richness did not decline to 50% of maximum (blue line, Figure 5a,b). Declines were 47% by 12 kg N and 44% by 20 kg S ha^−1^ y^−1^, our modeling endpoints. By our definition, this indicates that air pollution did not pose a high risk to the total biological diversity of epiphytic macrolichens in US forests from 1993–2012. By contrast, 90% quantiles generated by the deposition + climate model (fuzzed gray dots) showed that climate has a moderately large effect on species richness in the West. The first 5 kg of the deposition gradient includes survey sites ranging from the hot, arid Southwest (lowest values) to the cool, coastal Alaskan rainforests (highest values) and the continental northern Rocky Mountains, and thus encompassed a large climatic gradient over a short pollution gradient. Above 5 kg N or S, the climate influence is narrower and less variable, reflecting the less limiting moisture and temperature conditions among the eastern survey sites that make up the preponderance of those sites. Overall, species richness is a bigger concern in the East than the West, but this metric is less sensitive to pollution than other metrics. Critical loads were 3.5 kg N and 6.0 kg S ha^−1^ y^−1^ (Table 3) model fits were fair at R1 = 0.11 and 0.07, respectively (Table 2).

#### 3.2.2. Forage Lichen Diversity and Abundance

Forage lichen diversity and abundance dropped rapidly with increasing N and S deposition, declining by 80% by 10 kg N and 13 kg S ha^−1^ y^−1^ (Figure 6a,b). Compared to 90% quantiles for the species richness ‘deposition + climate’ model, climate was a less important source of variability for forage lichens, above 5 kg N or S. Forage lichens are a sensitive indicator of pollution and models should be nationally applicable except in hot, dry climates not suited to these species, such as the arid forests of the interior west in Arizona, New Mexico, Nevada, Utah, Colorado, and Wyoming. Forage lichens may occur in these states in high elevation mountain forests in bands corresponding with frequent fog or in other moist locations (See Figure 6d). Critical loads were 2.0 kg N and 2.6 kg S ha^−1^ y^−1^ (Table 3); the model fits were good at R1 = 0.26 and 0.19 (Table 2).

#### 3.2.3. Cyanolichen Diversity and Abundance

High diversity and abundance among cyanolichens in coastal Alaska were observed at low deposition. Multiple sites supported 7–10 abundant species (Figure 6c,d). However, maximum values along the 90% quantiles were only 10 and 13 (3–4 species), due to the tremendous number of low counts and zeroes in the data set. Cyanolichen abundance dropped rapidly with deposition (80% declines) by 6.6 kg N and 11 kg S ha^−1^ y^−1^. Climate is crucial for cyanolichens, and low abundance in the west is largely explainable by unsuitable climates. In contrast, low abundance in the east is likely explained by excessive deposition. In summary, cyanolichens provide a sensitive measure of air pollution in with locations with yearlong cool, moist climates, e.g., the Level 1 US EPA Ecoregions: West Coast Marine forests, Northern Forests, and much of the Eastern Temperate Forests [66]. Critical loads for cyanolichen diversity and abundance were 1.3 kg N and 2.3 kg S ha^−1^ y^−1^, the lowest of all the functional group metrics (Table 3); the model fit was good for N (R1 = 0.19) and fair for S (R1 = 0.08) (Table 2).

#### 3.2.4. Matrix Lichen Diversity and Abundance

There were no correlations between the matrix lichen metric and deposition for first-, second-, or third-degree polynomials (R1 = 0) over the deposition range in US forests. Due to the broad range of pollution sensitivities of species in this functional group, it is not a useful indicator of pollution risk in the US. In areas climatically unsuitable for forage lichens or cyanolichens, we recommend assessing pollution effects using the sensitive species metric, which includes sensitive matrix lichens.

### 3.3. Response of Rare Species to Deposition

Table 5 shows an example of how a manager could evaluate extirpation risk for individual species of concern within a particular area. It displays N and S deposition levels associated with 0, 20, 50 and 90% declines in the probability of detecting some nationally rare lichens. For these particular species, one can see that the risk of extirpation would be low as long as the deposition is < 4 kg N and < 2.5 kg S ha^−1^ y^−1^. However deposition above 17 kg N ha^−1^ y^−1^ and 24 kg S ha^−1^ y^−1^ would pose a very high risk of extirpation for all of the species and 9 kg ha^−1^ y^−1^ of either S or N would pose a very high risk of extirpation for about half the species. A similar exercise could be made for other species in [19]’s Table S2. See also Section 4.3.3.

### 3.4. National Patterns of Lichen Diversity and Abundance

**Figure 7.**
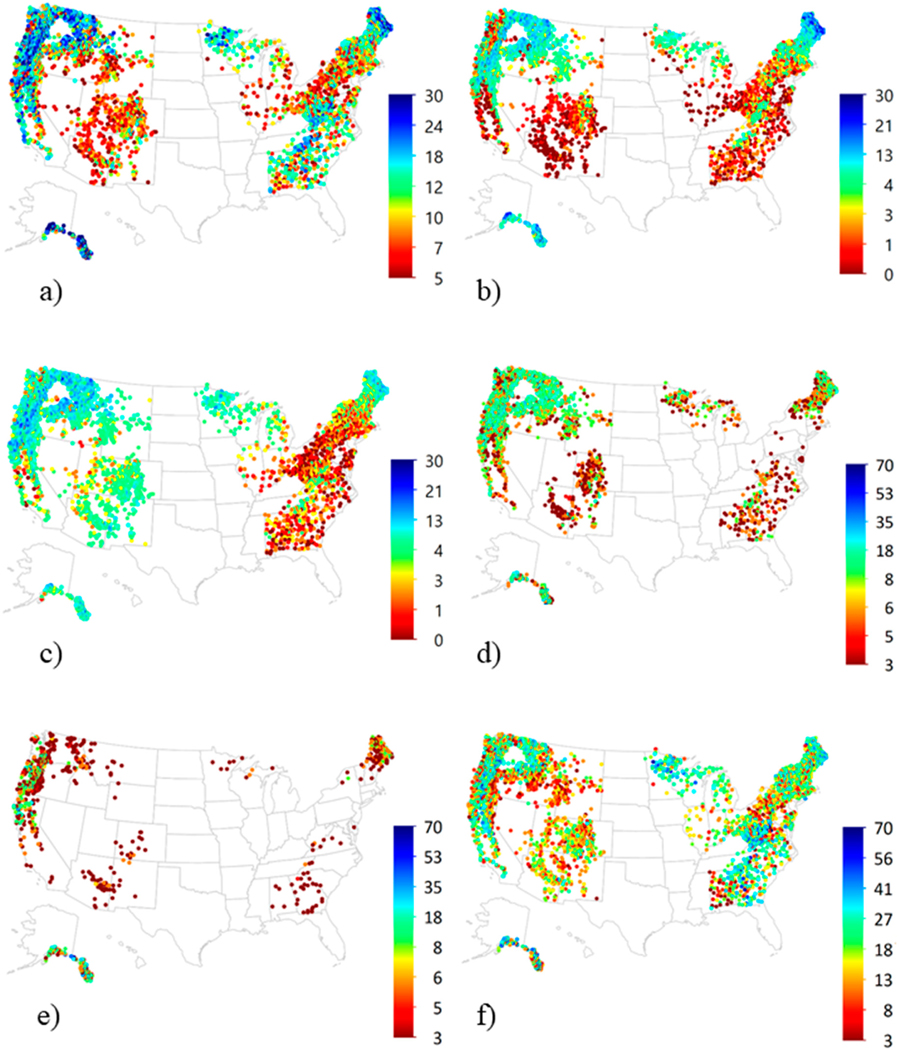
National maps of epiphytic macrolichen metrics for forests of the United States, 1990–2012: Species richness (count) of (**a**) all epiphytic macrolichens, (**b**) oligotrophs, (**c**) S-sensitive species; and diversity and abundance (sum ocular abundance ratings > 2) for (**d**) forage lichens, (**e**) cyanolichens and (**f**) matrix lichens. Sites with total species richness < 5 were not included in analyses.

National-scale maps of lichen metrics (Figure 7) were consistent with national pollution and climate gradients at the time of the surveys. Species richness was low throughout the mid-Atlantic and southern New England states with a long history of enhanced deposition, and across the comparatively hot, dry states of the Southwest US. Sulfur-sensitive species were widespread and dominated the flora of the West with localized impacts near major metropolitan areas. In contrast, S-sensitive species comprised a minor percentage of lichen communities throughout the East, consistent with the blanket acidification of precipitation there. Forage lichens were widespread and abundant in the Northwest, the Rocky Mountains south to western New Mexico and Colorado, the northern Great Lakes area (Minnesota, Wisconsin, and Michigan) and Maine. They were present in the Appalachian Mountains and southeastern Plains of the eastern US, but abundance was variable. Cyanolichens were most abundant in the coolest, wettest states with good air quality (Alaska, Pacific Coast, Minnesota, Wisconsin, Michigan, and Maine), but were absent or comprised <10% of the lichen community elsewhere.

## 4. Discussion

### 4.1. Summary of Results

Across US forests, sensitive species richness, forage lichen diversity and abundance and cyanolichen diversity and abundance were, on average, more sensitive to N and S deposition than total species richness. These lichen metrics were best predicted by total N or S deposition plus climate, with deposition contributing to the greater predictive power, as demonstrated by ΔAIC. Matrix lichen abundance and diversity was not responsive to deposition. Overall, lowest species richness and diversity and abundance of ecologically important forage and cyanolichen functional groups were observed in regions of the country with a long pollution history and regions with the warmest, driest climates. We demonstrated a method for calculating the risk of extirpation for species of conservation concern using depositions associated with 20, 50, and 90% declines in detection frequency from Table S2 in [19]. Rare species were no more likely to be air pollution sensitive than abundant species; declines of 20, 50 and 90% from maximum detection frequency were used to evaluate extirpation risk.

### 4.2. Estimating Risk

#### 4.2.1. Selecting Critical Loads

We selected a change of 20% from the maximum value for the modeled 90% quantile as the critical load for lichen diversity and abundance responses. This selection is based on the relative consistency of the measured responses above the 20% change cut-off and is consistent with our field observations of early harm. More explicitly, Figures 5 and 6 show that the maximal modeled response using 2nd degree polynomials always equaled the minimum deposition increment (i.e., 0.2 kg N and 0.2 kg S ha^−1^ y^−1^ in the West and 3.6 kg N and 2.2 kg S ha^−1^ y^−1^ in the East) whereas the actual responses tended to peak between modeled declines of 0 and 20% and to also range much higher above the predicted value below 20% change than above 20% change. We did test 3rd degree polynomials for all metrics without improvement in fit.

National critical loads for total species richness, sensitive species richness, and diversity and abundance of forage and cyanolichen functional groups were 3.5, 3.1, 1.9, and 1.3 kg N ha^−1^ y^−1^ and 6.0, 2.5, 2.6, and 2.3 kg S ha^−1^ y^−1^, respectively. We consider declines in metrics between 0 and 20%, >20–50%, >50–80% and >80% to be indicative of low, moderate, high and very high risk, respectively. Our primary considerations for selecting the 50−80% cut-offs included the degree to which species counts and abundances would be affected, the recovery time needed once air quality improves (Section 4.2.4), and potential effects on interdependent biota (Section 4.4). Deposition associated with each proposed risk level is presented in Table 3. However, decision-makers can use our regression equations (Figures 5 and 6) or Table 4 for selecting different target loads or risk classes that suit their own goals and opinions of what constitutes significant ecological harm.

#### 4.2.2. Comparison to Other Critical Loads

Our CLs compliment the national CLs proposed by [19] for lichen community composition in US forests (1.5 kg N and 2.7 kg S ha^−1^ y^−1^). The metrics from both studies can be used to assess critical loads exceedances without knowing which species occur in the analysis area, although we recommend the forage and cyanolichens critical loads be applied only in areas with suitable climates (see Sections 3.2.2 and 3.2.3). Our values are also compatible with lichen CLs determined from regional studies in the US [16], with many falling in the 1–4 kg N ha^−1^ y^−1^ range. We would not expect values to match exactly, however, as the metrics differ. Here, the response metrics were designed for evaluating air pollution’s risks to parameters directly linked to mission operational goals: Biodiversity, ecologically important functional groups, and species of conservation concern.

Our CLs for N (i.e., 1.3–3.5 kg N ha^−1^ y^−1^) are also consistent with the CL of 2.4 kg N ha^−1^ y^−1^ proposed recently by [67] for epiphytic macrolichen of European forests. Higher lichen CLs for Europe have been estimated at 5–15 kg N ha^−1^ y^−1^; but as N deposition has decreased, so have the CL estimates [17]. The paucity of natural background sites continues to hinder CL assessments in Europe and the eastern US. In a detailed assessment for Britain, [18] reported few observations of N deposition less than 5 kg N ha^−1^ y^−1^. They reported monotonically negative relationships for many ground-dwelling lichen species, suggesting their true CLs lie below the lowest observable deposition levels and that species with even lower CLs have potentially been lost.

To our knowledge, lichen critical loads for sulfur deposition have not been reported for lichens aside from this and our companion study [19]. There is, however, an extensive literature documenting lichen sensitivity to sulfur dioxide and acidic deposition (measured as pH) in Europe and the US [13,33,68,69]. Indeed, the first quantitative lichen-air quality index focused specifically on sulfur dioxide [70] and documented a range of tolerances among epiphytic species. We consider our S models as tools to measure lichen response to acidic deposition, as opposed to SO_2_ concentrations, which are currently below lichen response thresholds even in major US cities.

Our lichen CLs for N and S are comparable to acidification CLs in poorly buffered forest soils and sensitive water bodies [71,72] and much lower than some other receptors in U.S. ecosystems, including herbaceous species richness (8.7–13.4 kg N ha^−1^ y^−1^) [73], nitrate leaching (4–25 kg N ha^−1^ y^−1^) [16], and mycorrhizal fungi (5–12 kg N ha^−1^ y^−1^) [16]. However, the paradigm that lichens are the most sensitive vegetation is shifting. Recent research shows several species in other taxonomic groups are as sensitive as the sensitive lichens. For instance, many individual herb species have very low critical loads (e.g., < 2 kg N or S ha^−1^ y^−1^) [74]. [75]’s assessment of 71 tree species across the continental U.S. also found a wide variation in N and S sensitivities, with several decreasing at levels below 2 kg ha^−1^ y^−1^ of N or S. Yet, few lichens have tolerance levels as high as the tolerant herbs and trees, and therefore as a taxa group, they experience relatively higher ecological risk at high deposition.

#### 4.2.3. Number of Species Affected

The number of species affected at any given deposition varies greatly from location to location, dependent on deposition and other environmental factors, particularly climate, presence of hardwoods, and stand age [76]. Effects of increasing N or S deposition on the number of species detected (other environmental factors being suitable) along the 90th quantile for our national data set can be estimated from total and sensitive species counts provided in Table 5. For example, the 90% quantile model predicts a 27% decline in total species richness with 5 kg of N deposition. Therefore, if the 90% quantile is 33 species at 0.2 kg N, by 5 kg N species richness decreases by 9 species. One can estimate the variability around that optimal estimate, due to climate by visual inspection of the climate cloud in Figure 5, which at 5 kg of deposition is plus or minus about 5 species.

#### 4.2.4. Response Time Frames

Lichen community composition responds fairly quickly to changes in air quality. For example, nitrogen loving species can quickly propagate within a year or two while sensitive species may simultaneously or gradually succumb to suboptimal air quality, altered substrate pH, or drier, warmer climates [77–80]. Severe depletions of the flora were reported in the second half of the 20th century in the eastern US [33,81,82] related to high levels of acidifying and fertilizing S and N-containing air pollutants and in southern California [83] with high levels of fertilizing and oxidizing pollutants. Lichens do return when conditions are suitable; species of intermediate or high tolerance can return within a few years, but for other species the recovery may take decades [84–87], with higher cumulative emissions being associated with slower recovery rates [88]. Many large cyanolichens and pendant forage lichens are slow to disperse and are primarily associated with late-successional and old-growth forests [55,89]. Thus, the highest biomass of these functional groups requires not only good air quality but forest continuity of many decades, or even centuries.

Our data represent a snapshot in time. Plots in the East were surveyed from 1994–2005 (mean 1999.7, std. dev. 2.72). Western plots were surveyed from 1990–2012 (mean 2000.9, std. dev. 5.4). Between 2002 (when the bulk of the eastern lichen surveys had been completed) and 2017, concerted regulatory efforts have dramatically reduced deposition of total US emissions of NO_x_, SO_2_ and NH_4_ from 23,9599 to 10,770 and 12,217 to 2815 and 3994 to 3562 thousand tons, respectively [90]. Much of the improvement occurred in the East; the West has seen more moderate, but steady reductions in S deposition; and geographically specific increases or decreases in N deposition all of which are reflected in national deposition trends [91,92]. Resurveys of the original FIA protocol sites would be highly valuable for assessing recovery rates under different historical, spatial, and deposition improvement regimes. It could also help to fine-tune best intervals for deposition and climate explanatory variables for lichen response future modeling.

#### 4.2.5. Assessing Uncertainties

In the broad sense, empirical studies like this one do not measure cause and effect, and there are no large-scale deposition experiments in our study area to establish mechanistic responses by all the lichens. Likewise, our dataset covers many, but not all, possible combinations of climate + deposition, with narrower climate gradients in the East where deposition is highest. Interactions between pollutants, especially between N and S in the eastern US, the lack of clean sites in the East or high sulfur sites in the West, and uncertainties in the deposition estimates, species recovery rates, and species capture are main sources of uncertainty in our critical loads and risk analyses [16] and lichen biomonitoring analyses elsewhere [93].

That said, our CLs and response curves are based on an unprecedented amount of information. We used an exceptionally large community dataset (n = 8,855) and data interpretation draws from over 85 lichen-air quality studies conducted over the past 25 years using the FIA method. Our sensitivity ratings cover 362 species; about 63% of the taxa detected nationally. Thus, our values are strong starting points for understanding ecological risk as indicated by the novel lichen indices presented here. The similarities among the response functions in this study (Figures 5 and 6), their tight confidence intervals, and comparability of CLs with the other US and non-FIA CLs in Europe (Section 4.2.2) provide additional confidence that our estimates are reasonable.

### 4.3. Characterizing Risks to Lichen Biodiversity from Air Pollution

‘The most unique feature of Earth is the existence of life, and the most extraordinary feature of life is its diversity’ [94]. Human impacts are driving biodiversity losses at rates heralding a 6th mass extinction [95,96]. The US Endangered Species Act, the Convention on International Trade of Endangered Species of Wild Fauna and Flora (CITES) and the Western Hemisphere Convention are legal frameworks that require the conservation and protection of endangered and threatened species and their habitats. Aside from legal and ethical considerations, the impacts of diversity loss on ecological processes are real and appear poised to rival the impacts of other global drivers of environmental change [94,97,98].

#### 4.3.1. Total Species Richness at Community and Landscape Levels

The current North American lichen checklist north of Mexico [99] counts 4,786 species of lichens, 615 lichenicolous fungi (species living exclusively on lichen surfaces, in commensal to parasitic relationships with lichens) and 107 saprophytic fungi related to lichens. Approximately 1800 lichens have been detected in forested ecosystems of Alaska and the continental US during Forest Service sponsored surveys [100]. Worldwide the current lichen species estimate is about 22,000 species [101]. As seen in many studies, including this one, air pollution has detrimental effects on lichen species richness that can be readily measured. This metric lacks the nuance and responsiveness of functional group or community composition analysis. Ecologically valuable species may initially be replaced by less valuable species without changing total diversity, particularly in the case of nutrient N [20,22] Matrix lichens, with large numbers of tolerant species, dominate total species richness (54% in the West, 76% in the east), diluting the responsiveness of the metric. Nevertheless, measures of total diversity have value as a gauge of conservation success [97]. Maintaining biodiversity is a common management goal and species richness is easy to understand. If deposition is less than 3.5 kg N or 6 kg S ha^−1^ y^−1^, there is a low risk of harm to total species diversity of epiphytic macrolichens.

#### 4.3.2. Sensitive Species and Functional Groups

As we saw, certain groups of species (forage, cyanolichens, oligotrophs, S-sensitives) are more vulnerable than others. Functional groups with species encompassing a broad range of climate and pollution tolerances, such as the matrix lichens, are more robust. While all species (and ecological roles) may not be completely interchangeable within a group, we expect the wide range of species’ sensitivities helps buffer the ecological functions of matrix lichens from pollution effects. Matrix lichens play many general ecological roles: Habitat for insect populations in the canopy; nest decorating materials for hummingbirds, bushtits and other birds; and foraging habitat for insectivorous birds and predatory invertebrates [102].

For those species with specific ecological roles and high sensitivity, like forage and cyanolichens, air pollution is a concern because there are few to no tolerant species in these groups. In addition, climate warming can exacerbate effects of pollution, particularly for nitrogen-sensitive species and will therefore have larger impacts on forage lichens and cyanolichens that uniformly require cooler, moister environments than on matrix lichens as a group. Indeed, climate projections in the western US [103] combined with continued regulatory efforts to reduce pollution, indicate to us that hot, dry temperatures will soon become a more important driver of forage and cyanolichen declines than air pollution there. Besides the clear benefits to human and environmental health, another advantage of reducing air pollution is improved climate resiliency, for example of lichens [19]. Protecting the diversity of forage and cyanolichens was associated with deposition levels less than 1.3 to 2.0 kg N and 2.3 to 2.6 kg S ha^−1^ y^−1^. Many parts of the world [104], particularly in Asia and Europe, have deposition levels that pose a very high risk (> 6.6–10 kg N or >11–13 kg S ha^−1^ y^−1^) to these species.

Maintaining lichen diversity over broad ecological areas will help to prevent local extirpations of rare species, favor local diversity (species area curve), provide more consistent ecosystem functions and services and provide propagules for uphill movement of species with climate change.

#### 4.3.3. Rare Species

Rare species conservation is challenging because rare species defined here as species detected on <1% of survey sites, comprised about 56% of the total diversity of epiphytic macrolichens encompassed by US Forest Service surveys. These species have the most poorly understood habitat requirements, pollution sensitivities, and ecological roles. However rare species with rated pollution sensitivities were no more or less likely to be pollution sensitive than common species.

### 4.4. Characterizing Risks to Ecological Function and Integrity from Air Pollution

There is mounting evidence that biodiversity increases the stability of ecosystem functions through time while biodiversity loss contributes to accelerating declines in ecosystem processes [97]. Maintaining multiple ecosystem processes at multiple places and times requires higher levels of biodiversity than does a single process at a single place and time [94]. With lichens, we note that sites with plentiful matrix, forage, and cyanolichens provide more food, nesting materials, and suitable habitat for a greater number of species for lichenicolous fungi, insects, mollusks, other invertebrates, birds, small mammals, and ungulates, and even some reptiles and amphibians. As the cyanolichens and forage lichens decline with increasing deposition, one could expect impacts to their dependent biota.

#### 4.4.1. Forage Lichens

Forage lichens encompass the pendant beard and medium to large fruticose (shrubby) species (Table 1). They are widespread in mountainous areas, coastal habitats, and northern locations with cool moist climates and good air quality. They are widely utilized for forage by a variety of ungulates (deer, caribou, bison, moose, elk), rodents (flying squirrels, woodrats, voles), and lagomorphs (hares, marmots, pikas), particularly in older forests where they can dominate the lichen biomass. Small mammals and many bird species also use pendant lichens as nesting material.

Mammal dependence on forage lichens as food may be seasonal (winter) or year-round. They may serve as a mineral-rich dietary supplement, or like for the endangered woodland caribou, as a dominant component of their year-round diet. Lichen-dependent wildlife are, in turn, prey for other species. For example in parts of its range, the northern red-backed vole relies primarily on lichens as forage in winter [105] and is itself a staple of marten, fox, weasels, coyote, and snowy owls, comprising up to 74% of the diet of marten in some boreal forests [106,107]. The more exclusive the animal’s diet, the greater the risks posed by air pollution. For instance, the northern flying squirrels depend almost exclusively on the pendant forage lichen, *Bryoria*, in the winter while serving as the primary prey of the endangered northern spotted owl in the northern part of its range [108]. Thus, we would expect the loss of *Bryoria* would likely have cascading effects in this food web.

#### 4.4.2. Cyanolichens

Cyanolichens contribute significant new fixed nitrogen to old-growth temperate rain forests of the Pacific Northwest and southern coastal Alaska [109,110] where they can dominate the lichen biomass [111]. Large cyanolichens fill unique ecological roles as high quality, nutrient rich food for invertebrates and protective cover from desiccation and predation [55,112,113].

Risks to ecological processes related to cyanolichen decline are limited in geographical scope to areas with suitable climates (Figure 6): However, cyanolichen rich habitats can be important hotspots of biological diversity [114,115]. Because cyanolichens were among the most sensitive to air pollution and play unique roles, air pollution can readily diminish their contributions to forest integrity [111].

#### 4.4.3. Matrix Lichens

Matrix lichens comprised the remaining species; these are green algal foliose lichens of all sizes. As mentioned, they are, on average, more pollution tolerant than the other functional groups. There are more likely to continue to fulfill their varied ecological roles (see Table 1 and Section 4.4.2) under the range of deposition levels observed in US forests (up to 15 kg N and 30 kg S ha^−1^ y^−1^).

### 4.5. Characterizing Risks to Ecosystem Services from Air Pollution

Diversity becomes increasing important as a management goal at large geographic scales by providing a broader array of ecosystem services [97]. Loss of diversity across trophic levels, as may occur among co-dependent species, potentially influences ecosystem functions more strongly than diversity loss within a trophic level [94]. Further, as climate patterns change, organisms adapted to a particular suite of conditions must be able to migrate. Therefore maintaining the diversity of lichens and other species at large spatial scales not only favors higher diversity at local scales (according to species area curves), but also helps create resilience to climate change [116]. Five examples of ecosystem services affected by the loss of lichens are described below.

#### 4.5.1. Food, Fiber, Hunting, Recreation

Forests with high lichen cover provide important invertebrate foraging habitat for song birds: The rich protein source helps ensure reproductive success [56]. As these birds, e.g., the yellow warbler, *Setophaga petechia* [117] migrate south, they provide insect pest reduction services valued by agriculturalists. To the extent that birds and invertebrates depend on forage and cyanolichens, air pollution can affect their success in finding sufficient food or habitat.

Mycorrhizal fungi are obligate symbionts necessary to the growth of trees and other commercially important woody plants. Many ectomycorrhizal fungi rely on mycophagous small mammals, such as squirrels, chipmunks, voles, and mice for spore dispersal [118]. Many animals, both mycophages and predators, depend on trees for shelter, food and breeding places. As mentioned earlier, at least some species are obligate forage lichen consumers. Because mycorrhizae, air pollution sensitive lichens, and small mammals inseparably affect the structure, functioning, stability, and productivity of certain northwest forest ecosystems, loss of sensitive lichens, has implications for agricultural and silvicultural products.

Lichens, especially abundant showy macrolichens like the large cyanolichens, contribute to the aesthetic diversity, smells, and visual beauty of wilderness valued by hikers, outdoor enthusiasts, naturalists, and recreationists [111]. Ungulates like deer, caribou, elk, and moose that utilize lichens are sought in large numbers by subsistence and recreational hunters. Recreationists and hunters, in turn, provide direct economic value to the communities close to the natural areas that they visit, and elsewhere through their purchases of food, lodging, permits, equipment, vehicles, supplies, and souvenirs [119]. Loss of sensitive lichens and lichen diversity therefore has unquantified implications for subsistence and recreational services, as well as local and national economies.

#### 4.5.2. Pharmaceutical and Traditional Uses of Lichens

Traditional uses of lichens are part of the documented heritage of more than 100 indigenous tribes of North America. More than 300 species of lichens have traditional uses: Primarily for medicine [120], but also as food, dyes, fiber for bandages and bedding, textiles, and decorative materials [121–123]. Widely used species include forage species like *Usnea*, *Alectoria*, *Ramalina*, and *Bryoria*), wolf lichen (*Letharia*) and the larger cyanolichens (*Lobaria* and *Sticta*).

Modern biological technologies are enabling the synthesis of pharmaceutically active compounds from organisms, like lichens, that cannot be easily grown or harvested at commercial scales. The great majority of lichens produce one or more bio-active metabolites, many with antioxidant, antibacterial, antiviral, and anticancer activity [124,125]. Approximately 1050 unique lichen metabolites have been identified [126], most not known from any other organisms. Thus, lichen diversity encompasses a natural library of undeveloped pharmaceutical potential. Among currently available non-prescription products (e.g., antibiotic creams, tinctures, and deodorant) usnic acid from forage species of *Usnea* and *Alectoria*, is a most commonly used active ingredient. Loss of lichen biodiversity thus has implications for pharmaceutical development, over-the-counter medicines, personal products, and traditional practices.

## 5. Conclusions

Air pollution poses a major threat to human and environmental health in many parts of the world. Nitrogen and sulfur-containing pollutants deposit nutrient nitrogen and acidity to the environment that can harm natural ecosystems. Here we created 8 manager-relevant lichen metrics for quantifying the ecological risk from N and S deposition across US Forests. We modeled these metrics using 90% quantile regression to distill and quantify the effect of air pollution alone under otherwise suitable environmental and climatic conditions. N and S critical loads ranged from 1.3–3.5 kg N and 2.3–6.0 kg S ha^−1^ y^−1^ and suggested cut-offs for moderate and high levels of harm for each metric. We also demonstrated how to quantify deposition effects on the detection frequency of species of conservation concern. Preventing exceedance of lichen CLs can help managers and regulators meet mission operational goals to protect biodiversity and sustain the health and productivity of forests. Protecting lichens also supports direct and indirect ecosystem services related to food, fiber, hunting, recreation, pharmaceuticals and traditional uses.

## Supplementary Material

Supplement1

## Figures and Tables

**Table 1.** Functional group ecological roles and affiliated lichen genera.

Fxl Group	General Ecological Roles	Genera Assigned to This Functional Group ^1^
Large cyano-lichens	Primary nitrogen-fixing epiphytes achieving high biomass in moist, temperate, old-growth forests, contributing significant amounts of new nitrogen to the forest floor. Nutrient-rich food for mollusks and other invertebrates; habitat and cover for invertebrates.	*Anomolobaria, Dendriscosticta, Lobaria, Nephroma, Peltigera, Pseudocyphellaria, Sticta*
Small to medium cyano-lichens	Nitrogen-fixing lichens, but typically low biomass, due to the small size and low abundance. Habitat and nutrient rich food for invertebrates.	*Collema, Dendriscocaulon, Enchylium, Erioderma, Fuscopannaria, Lathagrium, Leioderma, Leptogium, Leptochidium, Pannaria, Scytinium, Vahliella*
Pendant forage lichens	Critical winter forage for ungulates in areas with deep snow; primary winter forage for flying squirrels, voles, other rodents. Nesting materials for rodents and birds. Habitat and food for invertebrates.	*Alectoria, Bryocaulon, Bryoria, Nodobryoria,* pendant *Ramalina* and *Usnea*
Shrubby forage lichens	Winter forage for flying squirrels, voles, other rodents. Nesting materials for birds. Habitat and food for invertebrates.	*Bunodophorun, Evernia, Letharia, Pseudevernia, Sphaerophorus, Teleoschistes,* shrubby *Ramalina* and *Usnea*
Medium to large matrix lichens	Nesting materials for birds; habitat, cover and food for invertebrates.	*Ahtiana, Canoparmelia, Cetrelia, Crespoa, Esslingeriana, Flavoparmelia, Flavopunctelia, Heterodermia, Hypogymnia, Hypotrachyna, Imshaugia, Melanelixia, Melanohalea, Menegazzia, Montanelia, Myelochroa, Niebla, Parmelia, Parmelina, Parmotrema, Physcia, Physconia, Platismatia, Punctelia, Teloschistes, Tuckermanella, Tuckermannopsis, Usnocetraria, Vulpicida*
Small matrix lichens	Exposed habitat and food for invertebrates	*Anaptychia, Bulbothrix, Candelaria, Cavernularia, Cladonia, Coccocarpia, Crespoa, Hyperphyscia, Kaernefeltia, Loxosporopsis, Parmeliella, Parmeliopsis, Phaeophyscia, Physciella, Placidium, Polycaulon, Polychidium, Pyxine, Rusavskia, Xanthomendoza, Xanthoria*

1 See Discussion for references supporting ecological roles. Not all genera have been documented to play the roles associated with their assigned group. For species-level epithets, see Table S1 in [19] or [30].

**Table 2.** Goodness of fit statistics and improvements in AIC for 90% quantile regression models demonstrate a higher predictive value of deposition with climate compared to climate alone. Smaller AICs indicate better fit (ΔAIC must be >25). The larger the ΔAIC, the more improvement in the model compared to other models for that metric. R1 measures absolute model fit whereas AIC measures the relative fit of nested models (i.e., models in the same row). Model predictor variables: Dep = deposition only, Clim = all climate variables only, Dep + Clim = deposition and all climate variables.

	Dep ^1^		Clim		Dep + Clim ^2^		(Dep + Clim) − Clim
	
Lichen Metric	R1	AIC	R1	AIC	R1	AIC	Δ AIC^2^
Nitrogen Models							

Species richness	0.11	33265	0.09	33425	0.16	32807	619
Oligotroph	0.19	28672	0.11	29520	0.26	27887	1633
Cyanolichens	0.19	29465	0.21	29202	0.28	28419	783
Forage lichens	0.26	31982	0.14	33291	0.29	31626	1665

Sulfur Models							

Species richness	0.07	40123	0.07	39997	0.13	39308	689
Sensitive	0.23	32588	0.02	35055	0.25	32340	2715
Cyanolichens	0.08	37265	0.16	36297	0.22	35560	737
Forage lichens	0.19	39312	0.11	39562	0.23	38026	1536

1 Models used to assess risk.

2 Models used to explore the behavior of the metric under variable (instead of optimized) climate.

**Table 3.** Risks to lichen diversity and abundance, tabulated as the percent change in the 90% quantiles of lichen metrics associated with various levels of deposition, and recommended critical loads ^1^ (grey shading). Recommended risk categories: Low = < 20% decline in a metric, moderate = >20–50%, high = > 50–80%, and very high = > 80% declines. – = insufficient data to model.

	Deposition Yielding % Decline in Count or Abundance ^2^					Count or Abundance at % Decline from Maximum				

Lichen metric decline (%):	0 %	10 %	20 %	50 %	80 %	0 %	10 %	20 %	50 %	80 %
Nitrogen deposition										

Species richness	0.1	1.7	**3.5**	–	–	33	29	26	–	–
Oligotroph richness	0.1	1.5	**3.1**	8.3	14.8	18	16	15	9	4
Forage lichen abundance	0.1	1.0	**1.9**	5.3	10.4	30	27	24	15	6
Cyanolichen abundance	0.1	0.7	**1.3**	3.5	6.6	13	12	11	7	3

Sulfur deposition										

Species richness	0.2	2.9	**6.0**	–	–	29	26	23	–	–
S-sensitive species richness	0.2	1.3	**2.5**	6.7	14.1	17	16	14	9	3
Forage lichen abundance	0.2	1.4	**2.6**	6.9	13	24	21	19	12	5
Cyanolichen abundance	0.2	1.2	**2.3**	5.9	11	10	9	8	5	2

1 Boot-strapped 95% confidence intervals for the eight critical loads, from top to bottom are: 3.25–3.90, 2.77–3.38, 1.55–2.18, 0.49– 1.3, 5.39–6.81, 2.3–2.65, 2.25–2.78, and 1.81–2.77 kg ha^−1^ y^−1^.

2 Abundance = diversity and abundance metric= sum of ocular abundance ratings of 3 and 4 for the species detected.

**Table 4.** Predicted percent declines in lichen metrics with increasing nitrogen and sulfur deposition. - = not modeled, insufficient data.

Lichen Metric	Percent Decline in Metric											

Nitrogen (kg ha^−1^ y^−1^)	1	1.5	2	2.5	3	5	7.5	10	12.5	15	17.5	20
Species richness	6	9	12	15	17	27	37	44	48	49	-	
Oligotroph richness	6	10	13	16	19	32	46	59	70	81	90	97
Forage lichen abundance ^1^	10	16	21	26	31	48	66	78	87	90	-	-
Cyanolichen abundance ^1^	15	23	30	37	44	66	86	98	100	-	-	-

**Sulfur (kg ha**^−1^ **y**^−1^**)**	1	1.5	2	2.5	3	5	7.5	10	12.5	15	17.5	20

Species richness	3	5	7	9	10	17	24	31	36	40	43	45
S-sensitive species richness	7	12	16	20	24	39	55	67	76	82	84	-
Forage lichen abundance ^1^	7	11	15	19	23	38	54	68	79	88	94	98
Cyanolichen abundance ^1^	8	13	18	22	27	44	62	76	87	95	99	100

1 abundance = diversity and abundance metric= sum of ocular abundance ratings of 3 and 4 for the species detected.

**Table 5.** Deposition (kg ha^−1^ y^−1^) associated with 0, 20%, 50% and 90% declines in species occurrences can be used to quantify the risk of extirpation for rare species from eutrophying or acidifying air pollutants. Species of rare lichens were randomly selected from the national dataset to represent each pollutant (Poll), sensitivity class (Sensitivity), area, and functional group (Fxl Gp). The last four column headings are the percentage decrease from maximum detection frequency from Table S2 in [19]; values are deposition in kg ha^−1^ yr^−1^. Risk of extirpation is proposed as low at deposition levels associated with declines of 0–20%, moderate from 20–50%, high from 50–90% and very high for declines over 90%. Abbreviations: Oligo = oligotroph, meso = mesotroph, eut= eutroph, sens = sensitive, intm = intermediate, tol = tolerant, occ = occurrences, i.e. The number of times species was detected in the Area. E = eastern US (2156 survey sites), W = western US (6699 survey sites). Fxl Gp abbreviations as in Figure 3.

				Risk:		Low 0–20%		Hi 50–90%	
						
							Mod 20–50%		V. hi >90%

Rare Lichen	Poll	Sensi-tivity	Area	Fxl Gp	occ	0	−20%	−50%	−90%
*Nephroma occultum*	N	oligo	W	Cya l	29	2.9	4	4.9	6
*Pannaria conoplea*	N	oligo	E	Cya s-m	14	4.2	5.7	7	8.8
*Collema subflaccidum*	N	meso	W	Cya s-m	9	4.6	5.8	6.5	12.3
*Ramalina sinensis*	N	meso	E	For sh	18	7.0	8.2	9.2	11
*Heterodermia leucomela*	N	eut	W	Mtx m-l	36	8.2	9.9	11	12.2
*Coccocarpia palmicola*	N	eut	E	Mtx s	13	9.9	11.6	14	17

*Scytinium cellulosum*	S	sens	W	Cya s-m	13	1.6	2.5	3.2	8.8
*Usnea longissima*	S	sens	E	For p	18	2.7	4.7	5.8	7.6
*Ramalina obtusata*	S	intm	W	For sh	9	5.3	6.8	7.7	8.7
*Pseudocyphellaria crocata*	S	intm	E	Cya l	15	4.2	5.4	6.5	8.2
*Cladonia cenotea*	S	tol	W	Mtx s	29	8.7			
*Heterodermia granulifera*	S	tol	E	Mtx m-l	15	13.8	16	18.8	23.9
