# Supplementary material for: Assessing Ecological Risks from Atmospheric Deposition of Nitrogen and Sulfur to US Forests Using Epiphytic Macrolichens
Source: Diversity (Basel). Author manuscript; Available in PMC 2021 Oct 27. (PMC8549857; doi:10.3390/d11060087)
Supplement: Supplement1 [file NIHMS1732714-supplement-Supplement1.pdf]

Supplementary Materials

Table S1. Rare species sensitivity rating means (kg ha^{-1} y^{-1}) did not differ from common species except for S deposition in the West where rare species means and variances were a little lower (Analysis of Means transformed ranks; $p \leq 0.05$).

Pollutant	Area	Group	Group n	Lower Limit	Group Mean	Upper Limit	Limit Exceeded
N	East	common	25	0.530	0.703	1.058	
N	East	rare	49	0.621	0.736	0.966	
N	West	common	41	0.588	0.806	0.997	
N	West	rare	101	0.682	0.739	0.903	
S	East	common	26	0.547	0.868	1.047	
S	East	rare	54	0.642	0.665	0.952	
S	West	common	40	0.589	0.730	0.981	
S	West	rare	119	0.694	0.669	0.877	Lower

Table S2. Rare species sensitivity rating variances (kg ha^{-1} y^{-1}) did not differ from common species except for S deposition in the West where rare species means and variances were a little lower (Analysis of means for ADM-Leven Variances; $p \leq 0.05$).

Pollutant	Area	Group	Group n	Lower Limit	Group Mean ADM	Upper Limit	Limit Exceeded
N	East	common	25	2.287	3.113	4.459	
N	East	rare	49	2.665	3.037	4.081	
N	West	common	41	2.270	2.742	3.750	
N	West	rare	101	2.612	2.717	3.408	
S	East	common	26	2.596	5.759	8.059	
S	East	rare	54	3.632	4.359	7.023	
S	West	common	40	1.203	2.803	2.671	Upper
S	West	rare	119	1.596	1.438	2.278	Lower

Table S3. Pearson's correlations between predictors and variance inflation factors for Nitrogen models.

	N	Maxaug_c	Mindec_c	Precip_cm	Continent	CMD
**N**	1	0.48	0.15	−0.15	0.25	−0.1
**maxaug_c**	0.48	1	0.32	−0.54	0.52	0.53
**mindec_c**	0.15	0.32	1	0.36	−0.64	0.12
**precip_cm**	−0.15	−0.54	0.36	1	−0.76	−0.5
**continent**	0.25	0.52	−0.64	−0.76	1	0.32
**CMD**	−0.1	0.53	0.12	-0.5	0.32	1
**VIF**	NA	2.53	2.1	2.92	--[1]	1.6

[1] continentality is calculated using Aug and Dec temperature and therefore is perfectly correlated.

Table S4. Pearson's correlations between predictors and variance inflation factors for Sulfur models.

	S	Maxaug_c	Mindec_c	Precip_cm	Continen	CMD
**S**	1	0.31	0.07	−0.08	0.18	−0.2
**maxaug_c**	0.31	1	0.29	−0.53	0.51	0.54
**mindec_c**	0.07	0.29	1	0.39	−0.67	0.1
**precip_cm**	−0.08	−0.53	0.39	1	−0.76	−0.5
**continen**	0.18	0.51	−0.67	−0.76	1	0.32
**CMD**	−0.2	0.54	0.1	−0.5	0.32	1
**VIF**	NA	2.43	2.07	2.88	--[1]	1.6

[1] continentality is calculated using Aug and Dec temperature and therefore is perfectly correlated.
